# *N*-(2-Oxo-2-phenyl­acet­yl)benzamide

**DOI:** 10.1107/S160053681005258X

**Published:** 2010-12-18

**Authors:** Hoong-Kun Fun, Jia Hao Goh, Dongdong Wu, Yan Zhang

**Affiliations:** a X-ray Crystallography Unit, School of Physics, Universiti Sains Malaysia, 11800 USM, Penang, Malaysia; b School of Chemistry and Chemical Engineering, Nanjing University, Nanjing 210093, People’s Republic of China

## Abstract

In the title compound, C_15_H_11_NO_3_, the two essentially planar benzaldehyde groups [maximum deviations = 0.0487 (12) and 0.0205 (10) Å] are inclined at a dihedral angle of 72.64 (6)° with respect to each other. The bridging C—C—N—C torsion angle is 22.58 (18)°. In the crystal, inter­molecular bifurcated acceptor N—H⋯O and C—H⋯O hydrogen bonds link inversion-related mol­ecules into dimers incorporating *R*_1_^2^(7) and *R*_2_^2^(8) ring motifs. The crystal structure is further stabilized by weak inter­molecular C—H⋯π inter­actions.

## Related literature

For general background to and applications of the title benzamide compound, see: Haffner & Ulrich (2010 ▶); Lavanya & Rao (2010 ▶); Magarl *et al.* (2010 ▶). For graph-set descriptions of hydrogen-bond ring motifs, see: Bernstein *et al.* (1995 ▶). For related benzamide structures, see: Jotani *et al.* (2010 ▶); Fu *et al.* (1998 ▶); Gallagher *et al.* (2009 ▶). For related diketone structures, see: Cheah *et al.* (2008 ▶); Hartung *et al.* (2004 ▶). For standard bond-length data, see: Allen *et al.* (1987 ▶).
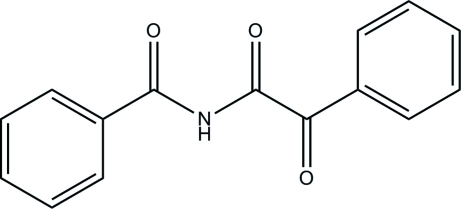


## Experimental

### 

#### Crystal data

C_15_H_11_NO_3_
*M* *_r_* = 253.25
Monoclinic, 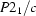

*a* = 5.7215 (1) Å
*b* = 10.7241 (1) Å
*c* = 20.6710 (3) Å
β = 98.255 (1)°
*V* = 1255.19 (3) Å^3^
*Z* = 4
Mo *K*α radiation
μ = 0.09 mm^−1^
*T* = 293 K
0.36 × 0.33 × 0.27 mm

#### Data collection

Bruker SMART APEXII CCD area-detector diffractometer
Absorption correction: multi-scan (*SADABS*; Bruker, 2009 ▶) *T*_min_ = 0.967, *T*_max_ = 0.975
13904 measured reflections
3624 independent reflections
2549 reflections with *I* > 2σ(*I*)
*R*_int_ = 0.025

#### Refinement

*R*[*F* ^2^ > 2σ(*F* ^2^)] = 0.044
*wR*(*F* ^2^) = 0.124
*S* = 1.03
3624 reflections
176 parameters
H atoms treated by a mixture of independent and constrained refinement
Δρ_max_ = 0.22 e Å^−3^
Δρ_min_ = −0.19 e Å^−3^

### 

Data collection: *APEX2* (Bruker, 2009 ▶); cell refinement: *SAINT* (Bruker, 2009 ▶); data reduction: *SAINT*; program(s) used to solve structure: *SHELXTL* (Sheldrick, 2008 ▶); program(s) used to refine structure: *SHELXTL*; molecular graphics: *SHELXTL*; software used to prepare material for publication: *SHELXTL* and *PLATON* (Spek, 2009 ▶).

## Supplementary Material

Crystal structure: contains datablocks global, I. DOI: 10.1107/S160053681005258X/lh5192sup1.cif

Structure factors: contains datablocks I. DOI: 10.1107/S160053681005258X/lh5192Isup2.hkl

Additional supplementary materials:  crystallographic information; 3D view; checkCIF report

## Figures and Tables

**Table 1:** Hydrogen-bond geometry (Å, °) *Cg*1 is the centroid of the C1–C6 benzene ring.

*D*—H⋯*A*	*D*—H	H⋯*A*	*D*⋯*A*	*D*—H⋯*A*
N1—H1*N*1⋯O2^i^	0.879 (16)	2.107 (16)	2.9765 (14)	170.0 (15)
C11—H11*A*⋯O2^i^	0.93	2.51	3.4080 (18)	162
C14—H14*A*⋯*Cg*1^ii^	0.93	2.86	3.6592 (18)	145

Symmetry codes: (i) 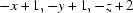; (ii) 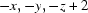.

**Fig. 1.**
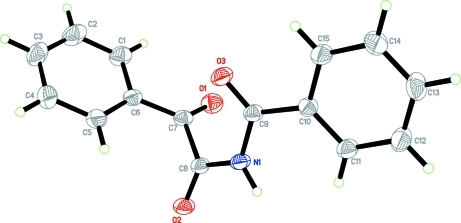
The molecular structure of the title compound, showing the atomic numbering scheme. Displacement ellipsoids for non-hydrogen atoms are drawn at the 30 % probability level.

**Fig. 2.**
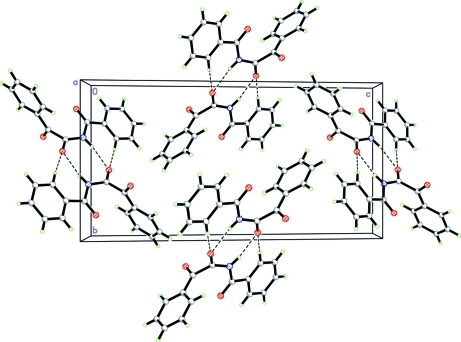
The crystal packing of the title compound, viewed along the a axis, showing hydrogen-bonded dimers lying parallel to the bc plane. Intermolecular hydrogen bonds are shown as dashed lines.

## supplementary crystallographic information

### Comment

Benzamides have been reported to correlate with many pharmacology processes such as anti-emetic, anti-psychotic and anti-arrythmic activities. Various *N*-substituted derivatives of benzamide are reported to possess anti-convulsant activity (Magarl *et al.*, 2010). Recently, Haffner & Ulrich (2010) reported that some *N*-substituted derivatives of benzamide can block the Kv1.3 ion channel. Moreover, *N*-substituted benzamides have been scanned for anti-microbial and anti-oxidant activities (Lavanya & Rao, 2010). The crystal structures of *N*-(2-oxo-2*H*-chromen-3-yl)benzamide (Jotani *et al.*, 2010), *N*-phenyl-*N*-(phenylthioxomethyl)benzamide (Fu *et al.*, 1998) and 2-fluoro-*N*-(2-fluorobenzoyl)-*N*-(2-pyridyl)benzamide (Gallagher *et al.*, 2009) have been reported. The title compound which contains the *N*-substituted benzamide has a potential use in biochemical and pharmaceutical fields. Due to the importance of the *N*-substituted benzamide derivatives, we report here the crystal structure of the title compound.

In the title compound (Fig. 1), the two benzaldehyde moieties (C1-C7/O1 and C9-C15/O3) are essentially planar, with maximum deviations of -0.0483 (12) Å at atom C7 and -0.0205 (10) Å at atom O3, respectively. The whole molecule is not planar, as indicated by the C7–C8–N1–C9 torsion angle of 22.58 (18)° and the dihedral angle formed between the two benzaldehyde moieties of 72.64 (6)°. The diketonic C7—C8 bond length [1.5401 (16) Å] is observed to be longer than expected value for a hybridized C*sp*^2^—C*sp*^2^ bond (Allen *et al.*, 1987), and is consistent to those observed in related diketone structures (Cheah *et al.*, 2008; Hartung *et al.*, 2004). All other geometrical parameters are comparable to those related *N*-substituted benzamide structures (Jotani *et al.*, 2010; Fu *et al.*, 1998; Gallagher *et al.*, 2009).

In the crystal structure, intermolecular bifurcated acceptor N1—H1N1···O2^i^ and C11—H11A···O2^i^ hydrogen bonds (Table 1) link inversion-related molecules into hydrogen-bonded dimers incorporating *R*^2^_1_(7) and *R*^2^_2_(8) ring motifs (Fig. 2, Bernstein *et al.*, 1995). Further stabilization of the crystal structure is provided by weak intermolecular C14—H14A···*Cg*1^ii^ interactions (Table 1) involving the centroid of the C1-C6 benzene ring.

### Experimental

The title compound was obtained in the photoreaction of 2,5-diphenyloxazole in visible light. The compound was purified by flash column chromatography. Good quality single crystals suitable for X-ray analysis were obtained from slow evaporation of a 1:1 solution of acetone and petroleum ether.

### Refinement

Atom H1N1 was located in a difference Fourier map and allowed to refine freely [N1—H1N1 = 0.879 (16) Å]. The remaining H atoms were placed in calculated positions, with C—H = 0.93 Å, and refined using a riding-model, with *U*_iso_(H) = 1.2 *U*_eq_(C).

### Crystal data

**Table:**

C_15_H_11_NO_3_	*F*(000) = 528
*M**_r_* = 253.25	*D*_x_ = 1.340 Mg m^−^^3^
Monoclinic, *P*2_1_/*c*	Mo *K*α radiation, λ = 0.71073 Å
Hall symbol: -P 2ybc	Cell parameters from 3981 reflections
*a* = 5.7215 (1) Å	θ = 2.8–28.7°
*b* = 10.7241 (1) Å	µ = 0.09 mm^−^^1^
*c* = 20.6710 (3) Å	*T* = 293 K
β = 98.255 (1)°	Block, colourless
*V* = 1255.19 (3) Å^3^	0.36 × 0.33 × 0.27 mm
*Z* = 4	

### Data collection

**Table:**

Bruker SMART APEXII CCD area-detector diffractometer	3624 independent reflections
Radiation source: fine-focus sealed tube	2549 reflections with *I* > 2σ(*I*)
graphite	*R*_int_ = 0.025
φ and ω scans	θ_max_ = 30.1°, θ_min_ = 2.0°
Absorption correction: multi-scan (*SADABS*; Bruker, 2009)	*h* = −8→7
*T*_min_ = 0.967, *T*_max_ = 0.975	*k* = −15→14
13904 measured reflections	*l* = −28→29

### Refinement

**Table:**

Refinement on *F*^2^	Primary atom site location: structure-invariant direct methods
Least-squares matrix: full	Secondary atom site location: difference Fourier map
*R*[*F*^2^ > 2σ(*F*^2^)] = 0.044	Hydrogen site location: inferred from neighbouring sites
*wR*(*F*^2^) = 0.124	H atoms treated by a mixture of independent and constrained refinement
*S* = 1.03	*w* = 1/[σ^2^(*F*_o_^2^) + (0.0558*P*)^2^ + 0.1732*P*] where *P* = (*F*_o_^2^ + 2*F*_c_^2^)/3
3624 reflections	(Δ/σ)_max_ = 0.001
176 parameters	Δρ_max_ = 0.22 e Å^−^^3^
0 restraints	Δρ_min_ = −0.19 e Å^−^^3^

### Special details

**Table:**

Geometry. All esds (except the esd in the dihedral angle between two l.s. planes) are estimated using the full covariance matrix. The cell esds are taken into account individually in the estimation of esds in distances, angles and torsion angles; correlations between esds in cell parameters are only used when they are defined by crystal symmetry. An approximate (isotropic) treatment of cell esds is used for estimating esds involving l.s. planes.
Refinement. Refinement of F^2^ against ALL reflections. The weighted R-factor wR and goodness of fit S are based on F^2^, conventional R-factors R are based on F, with F set to zero for negative F^2^. The threshold expression of F^2^ > 2sigma(F^2^) is used only for calculating R-factors(gt) etc. and is not relevant to the choice of reflections for refinement. R-factors based on F^2^ are statistically about twice as large as those based on F, and R- factors based on ALL data will be even larger.

### Fractional atomic coordinates and isotropic or equivalent isotropic displacement parameters (Å^2^)

**Table:**

	*x*	*y*	*z*	*U*_iso_*/*U*_eq_	
O1	0.09882 (19)	0.34229 (9)	0.84139 (5)	0.0559 (3)	
O2	0.55891 (18)	0.43948 (9)	0.92521 (5)	0.0541 (3)	
O3	0.15170 (19)	0.15978 (8)	0.95510 (5)	0.0526 (3)	
N1	0.2998 (2)	0.34922 (9)	0.98421 (5)	0.0425 (3)	
C1	0.3026 (3)	0.14025 (14)	0.78023 (7)	0.0555 (4)	
H1A	0.1544	0.1668	0.7607	0.067*	
C2	0.4136 (4)	0.04282 (16)	0.75346 (8)	0.0682 (5)	
H2A	0.3393	0.0029	0.7161	0.082*	
C3	0.6340 (3)	0.00455 (15)	0.78184 (8)	0.0658 (5)	
H3A	0.7078	−0.0613	0.7636	0.079*	
C4	0.7464 (3)	0.06306 (15)	0.83711 (9)	0.0632 (4)	
H4A	0.8964	0.0375	0.8557	0.076*	
C5	0.6352 (3)	0.15980 (13)	0.86475 (7)	0.0520 (3)	
H5A	0.7098	0.1988	0.9023	0.062*	
C6	0.4132 (2)	0.19867 (11)	0.83662 (6)	0.0409 (3)	
C7	0.2867 (2)	0.29847 (11)	0.86611 (6)	0.0399 (3)	
C8	0.4020 (2)	0.36431 (10)	0.92888 (6)	0.0401 (3)	
C9	0.1548 (2)	0.24758 (10)	0.99227 (6)	0.0387 (3)	
C10	0.0059 (2)	0.25238 (10)	1.04531 (6)	0.0383 (3)	
C11	−0.0018 (3)	0.35326 (11)	1.08721 (6)	0.0462 (3)	
H11A	0.0917	0.4230	1.0833	0.055*	
C12	−0.1486 (3)	0.34962 (13)	1.13475 (7)	0.0542 (4)	
H12A	−0.1524	0.4168	1.1630	0.065*	
C13	−0.2892 (3)	0.24711 (14)	1.14054 (8)	0.0612 (4)	
H13A	−0.3876	0.2453	1.1727	0.073*	
C14	−0.2843 (3)	0.14727 (14)	1.09876 (9)	0.0648 (4)	
H14A	−0.3802	0.0784	1.1024	0.078*	
C15	−0.1369 (3)	0.14978 (12)	1.05150 (7)	0.0527 (4)	
H15A	−0.1332	0.0821	1.0235	0.063*	
H1N1	0.337 (3)	0.4056 (15)	1.0148 (8)	0.055 (4)*	

### Atomic displacement parameters (Å^2^)

**Table:**

	*U*^11^	*U*^22^	*U*^33^	*U*^12^	*U*^13^	*U*^23^
O1	0.0567 (6)	0.0528 (6)	0.0559 (6)	−0.0008 (5)	0.0003 (5)	0.0004 (4)
O2	0.0593 (6)	0.0509 (5)	0.0563 (6)	−0.0261 (5)	0.0226 (5)	−0.0140 (4)
O3	0.0720 (7)	0.0380 (4)	0.0527 (5)	−0.0164 (4)	0.0258 (5)	−0.0117 (4)
N1	0.0522 (7)	0.0383 (5)	0.0390 (5)	−0.0163 (5)	0.0128 (5)	−0.0091 (4)
C1	0.0681 (10)	0.0593 (8)	0.0386 (6)	−0.0096 (7)	0.0057 (6)	−0.0075 (6)
C2	0.0970 (14)	0.0671 (10)	0.0431 (7)	−0.0139 (9)	0.0193 (8)	−0.0190 (7)
C3	0.0892 (13)	0.0541 (8)	0.0629 (9)	−0.0041 (8)	0.0409 (9)	−0.0113 (7)
C4	0.0576 (9)	0.0647 (9)	0.0715 (10)	0.0001 (7)	0.0234 (8)	−0.0067 (8)
C5	0.0504 (8)	0.0545 (8)	0.0522 (8)	−0.0094 (6)	0.0112 (6)	−0.0112 (6)
C6	0.0483 (7)	0.0412 (6)	0.0352 (6)	−0.0120 (5)	0.0129 (5)	−0.0029 (5)
C7	0.0466 (7)	0.0372 (6)	0.0366 (6)	−0.0123 (5)	0.0082 (5)	0.0006 (4)
C8	0.0454 (7)	0.0333 (5)	0.0431 (6)	−0.0090 (5)	0.0112 (5)	−0.0045 (4)
C9	0.0455 (7)	0.0334 (5)	0.0378 (6)	−0.0068 (5)	0.0077 (5)	−0.0012 (4)
C10	0.0444 (7)	0.0335 (5)	0.0380 (6)	−0.0031 (5)	0.0093 (5)	0.0024 (4)
C11	0.0559 (8)	0.0383 (6)	0.0459 (7)	−0.0074 (6)	0.0128 (6)	−0.0036 (5)
C12	0.0681 (10)	0.0472 (7)	0.0510 (8)	0.0018 (7)	0.0217 (7)	−0.0048 (6)
C13	0.0729 (10)	0.0553 (8)	0.0634 (9)	0.0031 (7)	0.0371 (8)	0.0073 (7)
C14	0.0778 (11)	0.0457 (7)	0.0793 (11)	−0.0143 (7)	0.0402 (9)	0.0038 (7)
C15	0.0675 (9)	0.0357 (6)	0.0598 (8)	−0.0096 (6)	0.0261 (7)	−0.0030 (5)

### Geometric parameters (Å, °)

**Table:**

O1—C7	1.2164 (16)	C5—H5A	0.9300
O2—C8	1.2170 (14)	C6—C7	1.4723 (18)
O3—C9	1.2138 (14)	C7—C8	1.5401 (16)
N1—C8	1.3667 (16)	C9—C10	1.4828 (17)
N1—C9	1.3943 (15)	C10—C15	1.3873 (17)
N1—H1N1	0.879 (16)	C10—C11	1.3904 (17)
C1—C2	1.379 (2)	C11—C12	1.3818 (19)
C1—C6	1.3929 (18)	C11—H11A	0.9300
C1—H1A	0.9300	C12—C13	1.378 (2)
C2—C3	1.375 (3)	C12—H12A	0.9300
C2—H2A	0.9300	C13—C14	1.378 (2)
C3—C4	1.379 (2)	C13—H13A	0.9300
C3—H3A	0.9300	C14—C15	1.380 (2)
C4—C5	1.383 (2)	C14—H14A	0.9300
C4—H4A	0.9300	C15—H15A	0.9300
C5—C6	1.383 (2)		
			
C8—N1—C9	121.74 (10)	O2—C8—N1	122.59 (11)
C8—N1—H1N1	115.6 (10)	O2—C8—C7	118.81 (11)
C9—N1—H1N1	122.6 (10)	N1—C8—C7	117.88 (10)
C2—C1—C6	119.74 (16)	O3—C9—N1	119.05 (11)
C2—C1—H1A	120.1	O3—C9—C10	122.50 (10)
C6—C1—H1A	120.1	N1—C9—C10	118.44 (10)
C3—C2—C1	120.14 (15)	C15—C10—C11	119.20 (12)
C3—C2—H2A	119.9	C15—C10—C9	116.69 (10)
C1—C2—H2A	119.9	C11—C10—C9	124.09 (11)
C2—C3—C4	120.52 (15)	C12—C11—C10	119.85 (12)
C2—C3—H3A	119.7	C12—C11—H11A	120.1
C4—C3—H3A	119.7	C10—C11—H11A	120.1
C3—C4—C5	119.75 (16)	C13—C12—C11	120.44 (13)
C3—C4—H4A	120.1	C13—C12—H12A	119.8
C5—C4—H4A	120.1	C11—C12—H12A	119.8
C4—C5—C6	120.10 (14)	C12—C13—C14	120.04 (14)
C4—C5—H5A	119.9	C12—C13—H13A	120.0
C6—C5—H5A	119.9	C14—C13—H13A	120.0
C5—C6—C1	119.73 (13)	C13—C14—C15	119.87 (13)
C5—C6—C7	121.43 (11)	C13—C14—H14A	120.1
C1—C6—C7	118.81 (13)	C15—C14—H14A	120.1
O1—C7—C6	124.36 (11)	C14—C15—C10	120.59 (13)
O1—C7—C8	115.00 (11)	C14—C15—H15A	119.7
C6—C7—C8	120.35 (11)	C10—C15—H15A	119.7
			
C6—C1—C2—C3	−0.8 (2)	O1—C7—C8—N1	71.15 (15)
C1—C2—C3—C4	−0.2 (3)	C6—C7—C8—N1	−114.71 (13)
C2—C3—C4—C5	0.9 (2)	C8—N1—C9—O3	13.09 (19)
C3—C4—C5—C6	−0.7 (2)	C8—N1—C9—C10	−165.59 (12)
C4—C5—C6—C1	−0.2 (2)	O3—C9—C10—C15	1.00 (19)
C4—C5—C6—C7	177.86 (13)	N1—C9—C10—C15	179.64 (12)
C2—C1—C6—C5	1.0 (2)	O3—C9—C10—C11	−177.49 (13)
C2—C1—C6—C7	−177.13 (13)	N1—C9—C10—C11	1.15 (19)
C5—C6—C7—O1	174.79 (12)	C15—C10—C11—C12	0.8 (2)
C1—C6—C7—O1	−7.10 (19)	C9—C10—C11—C12	179.20 (13)
C5—C6—C7—C8	1.23 (18)	C10—C11—C12—C13	−0.6 (2)
C1—C6—C7—C8	179.33 (11)	C11—C12—C13—C14	0.0 (3)
C9—N1—C8—O2	−167.27 (13)	C12—C13—C14—C15	0.5 (3)
C9—N1—C8—C7	22.58 (18)	C13—C14—C15—C10	−0.4 (3)
O1—C7—C8—O2	−99.38 (15)	C11—C10—C15—C14	−0.3 (2)
C6—C7—C8—O2	74.76 (16)	C9—C10—C15—C14	−178.82 (14)

### Hydrogen-bond geometry (Å, °)

**Table:**

Cg1 is the centroid of the C1–C6 benzene ring.

**Table:**

*D*—H···*A*	*D*—H	H···*A*	*D*···*A*	*D*—H···*A*
N1—H1N1···O2^i^	0.879 (16)	2.107 (16)	2.9765 (14)	170.0 (15)
C11—H11A···O2^i^	0.93	2.51	3.4080 (18)	162
C14—H14A···Cg1^ii^	0.93	2.86	3.6592 (18)	145

Symmetry codes: (i) −*x*+1, −*y*+1, −*z*+2; (ii) −*x*, −*y*, −*z*+2.
